# Comparative Evaluation of Cytotoxicity of Zinc Oxide Nanoparticles Mixed With Herbal Extract as an Intracanal Medicament: A Zebrafish Model Study

**DOI:** 10.7759/cureus.64131

**Published:** 2024-07-09

**Authors:** Atluri Manoj, Manish Ranjan, Sanyukta Singh, Chinnasamy Ragavendran

**Affiliations:** 1 Department of Conservative Dentistry and Endodontics, Saveetha Dental College and Hospitals, Saveetha Institute of Medical and Technical Sciences, Saveetha University, Chennai, IND; 2 Department of Cariology, Saveetha Dental College and Hospitals, Saveetha Institute of Medical and Technical Sciences, Saveetha University, Chennai, IND

**Keywords:** zinc oxide nanoparticles, zebrafish model, intracanal medicament, herbal extract, developmental toxicity, cytotoxicity

## Abstract

Objective

In this study, zebrafish embryos are used to study the cytotoxic effects of a novel intracanal medication (ICM) based on zinc oxide nanoparticles (ZnO NPs) loaded with polyherbal extracts (*Azadirachta indica *and *Solanum xanthocarpum*).

Material and methods

In the present study, a green and sustainable method was employed for the synthesis of ZnO NPs mixed with bark and seed extracts of *Azadirachta indica *and *Solanum xanthocarpum* to be used as a polyherbal ICM. Formulation of ZnO NPs was confirmed with color change in mixture produced upon dissolving zinc acetate dihydrate in distilled water followed by slow addition of sodium hydroxide solution and herbal extracts. The effects of these green synthesized ZnO NPs were evaluated through a zebrafish embryo toxicity test. Embryos were exposed to different concentrations (25, 50, and 100 µg/mL) of synthesized experimental doses of ZnO NP and compared with the control embryos. Toxicological endpoints, such as the zebrafish embryo’s survival rate, hatching rate, and heart rate, were noted and described.

Results

A concentration-dependent increase in mortality rate and hatching delay followed by declined heart rate was observed in green synthesized ZnO NP-treated embryos. The maximum toxicity was observed with an increase in the concentration of 100 µg/mL of the experimental dose, and at a low concentration of 25 µg/mL, it does not effectively show any developmental alteration in zebrafish embryos.

Conclusion

A novel polyherbal ICM loaded with ZnO NPs exhibited a dose-dependent effect on the heart rate, hatching, and mortality rate of the embryos. At optimal concentrations, the medication demonstrated minimal developmental malformations and cytotoxic effects, indicating its safety for use. However, increasing concentrations of the medication resulted in severe developmental malformations.

## Introduction

Successful endodontic therapy depends upon proper chemo-mechanical preparation, disinfection, and debridement, followed by optimum obturation [1]. Intracanal medicaments (ICMs) are instrumental in root canal therapy as they help create an environment conducive to periapical tissue repair by eliminating bacteria and reducing inflammation [2]. Factors such as the nature and extent of the infection and the overall treatment plan influence the choice and effectiveness of medicament. The dramatic increase of antibiotic-resistant bacteria and adverse reactions to synthetic drugs like calcium hydroxide as ICM necessitate effective substitutes [3,4].

Traditional medicine, along with phytoconstituents, has gained popularity due to its affordability, therapeutic value, and reputation for having fewer side effects than synthetic drugs [5]. Neem plant (*Azadirachta indica*), known for its antibacterial, antifungal, and antiviral properties [6], has a tannin-like substance in its bark that inhibits bacterial growth [7]. Kantakari (*Solanum xanthocarpum*), another common medicinal plant, is a commonly occurring perennial herb containing flavonoids, carbohydrates, tannins, and phenols that exhibit strong antibacterial properties against a variety of pathogens [8].

Research on nanotechnology is flourishing in the field of modern medical sciences. This technology may be used for many innovative purposes, such as advanced medical operations and food and agricultural production [5]. Through enhanced mechanical, physical, and diagnostic qualities as well as nano-delivery techniques, it has completely transformed the medical and dental professions [6]. Nanomaterials with diameters ranging from 1 to 100 nm are employed in place of other drug carriers for ease of mobility in the human body.

Zinc oxide nanoparticles (ZnO NPs) have been incorporated into a wide range of products such as cosmetics, toothpaste, textiles, etc. [9]. ZnO NPs can interact with bacteria's surface and/or core, leading to distinct bactericidal mechanisms at nanometer scales [10]. According to studies, ZnO NPs are nontoxic to human cells, making them useful as antibacterial agents, noxious to microorganisms, and biocompatible [8,11]. ZnO NPs are widely used as a versatile nanomaterial with therapeutic potential for a variety of diseases, but their therapeutic levels should be investigated [12].

The small tropical fish known as zebrafish, which is indigenous to South India, has gained popularity as a model organism in the fields of developmental genetics and nanomaterial ecotoxicology. [13]. Their suitability is attributed to a unique combination of features, including high genetic homology to humans, rapid development, ease of laboratory maintenance, prolific reproduction, transparency, and amenability to experimentation [14]. It is possible to create thousands of embryos every day in a laboratory environment and use them for multiple experiments at once. Zebrafish can generate thousands of embryos daily in laboratory settings, permitting simultaneous experimentation and rapid high-throughput screening of chemical and drug toxicity [15]. A number of pioneering studies have been conducted using zebrafish embryos to screen for novel drug candidates [16,17].

The aim of the present study is to assess the cytotoxic effects of novel polyherbal ICM containing ZnO NPs mixed with extract of *Azadirachta indica* bark and *Solanum xanthocarpum* seeds using an embryo zebrafish model.

## Materials and methods

Collection and authentication of plant specimens

*Azadirachta indica* bark was obtained from Guttoor Village, Kelamangalam, Krishnagiri District, and *Solanum xanthocarpum* seeds were collected near the bank of Cauvery River, Tamilnadu, from August to November 2023. A taxonomist from the Department of Botany at Periyar University, Salem, Tamil Nadu, India, authenticated the specimen. In order to conduct the experiment, zinc acetate dihydrate was purchased from Sigma-Aldrich Chemicals Pvt Ltd. (Merck Group, Bangalore, Karnataka, India).

Preparation of plant extracts

The bark of *Azadirachta indica* and seed extract of *Solanum xanthocarpum* were dried under shade and ground into a fine powder. A mixture of 10 g of fine powdered extract in 100 mL of distilled water was heated to 80°C for 25 minutes while being continuously stirred. As soon as the aqueous extracts were prepared, they were allowed to cool to room temperature and then filtered with Whatman No. 1 filter paper. The sterile extracts were stored at 4°C for further use.

Green synthesis of ZnO NPs

A 50 mL distilled water was dissolved in 2 mM zinc acetate dihydrate and kept on a stirrer for one hour. NaOH solution (20 mL) and 500 mL plant extracts were slowly added to the zinc acetate solution and stirred vigorously for three more hours at 60°C with a magnetic stirrer. After the mixture was mixed, it was allowed to stand for an entire night at room temperature. As a result of this procedure, a color change was observed in the mixture, confirming the synthesis of ZnO NPs. Separation of the residue from the reaction solution was carried out by centrifugal force at 15,000 rpm for 15 minutes, and pellets were collected. A hot air oven was used to dry the Petri plate before introducing the sediment/pellet mixture. A dryer was used to dry the plate for two days. After drying, the nano pellets were stored at 4°C in an airtight container.

Characterization of NPs

The synthesized ZnO NP maximum absorbance is measured using UV-visible (Vis) spectrophotometry (Model T80+UV-Vis spectrometer). ZnO NPs were investigated via ultraviolet and visible absorption spectroscopy in the range of 200-800 nm in order to determine their optical property. X-ray diffraction (XRD) was used to analyze the structure of ZnO NPs. Scanning electron microscopy (SEM) was used to measure the particle size of synthesized nanoparticles using a JEOLJEM-2100F SEM operated at 200 kV [18].

Zebrafish embryo toxicity assays

The male and female zebrafish were purchased from the local aquarium. The 60-L glass aquariums containing 20-L tap water are used to house the mature zebrafish. For the ideal aeration system, an aquarium foam sponge filter and an air pump are employed. Adult zebrafish were utilized for breeding, and embryos were collected and used for the following experiments (Ethical clearance number: SRB/SDC/ENDO-2204/24/113). After additional examination of the collected embryos under a microscope, the fertilized embryos are placed on a six-well plate and cultured in an E3 medium, while the unfertilized embryos are discarded. To evaluate acute toxicity and developmental defects caused by novel polyherbal ICM containing ZnO NPs, several test doses of ZnO NPs were administered to zebrafish embryos ranging from 25, 50, to 100 µg/mL calculated based on preliminary studies and compared to controls at any of the concentrations tested and observed for the presence of three endpoint parameters: mortality rate, hatching rate, and heart rate.

Statistical analysis

The findings were displayed as the triplicate mean alongside the standard deviation. Statistical analysis was carried out using GraphPad Prism software (Ver 5.03, CA, USA). One-way ANOVA was conducted to determine the significance levels between the groups (p < 0.05).

## Results

Effects on survival

**Figure 1:**
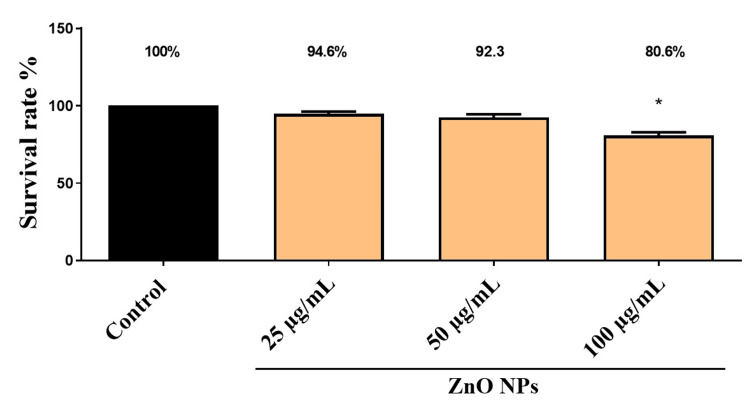
The percentage of survival rate of zebrafish embryos treated with various concentrations of synthesized ZnO NPs. The * (p < 0.05) indicates the significant difference between the control and treatment groups.

The mortality rate for synthesized ZnO NPs at various concentrations is shown in Figure 1. Controls had a 100% survival rate, while ZnO NP-treated groups showed similar survival rates at 25 and 50 µg/mL. The survival rate of zebrafish embryos declined when the concentration was 100 g/mL, leading to deformities in the tail and spinal curvature. Zebrafish embryos are more likely to die when concentration increases, according to this study.

Effects on heart rate

**Figure 2:**
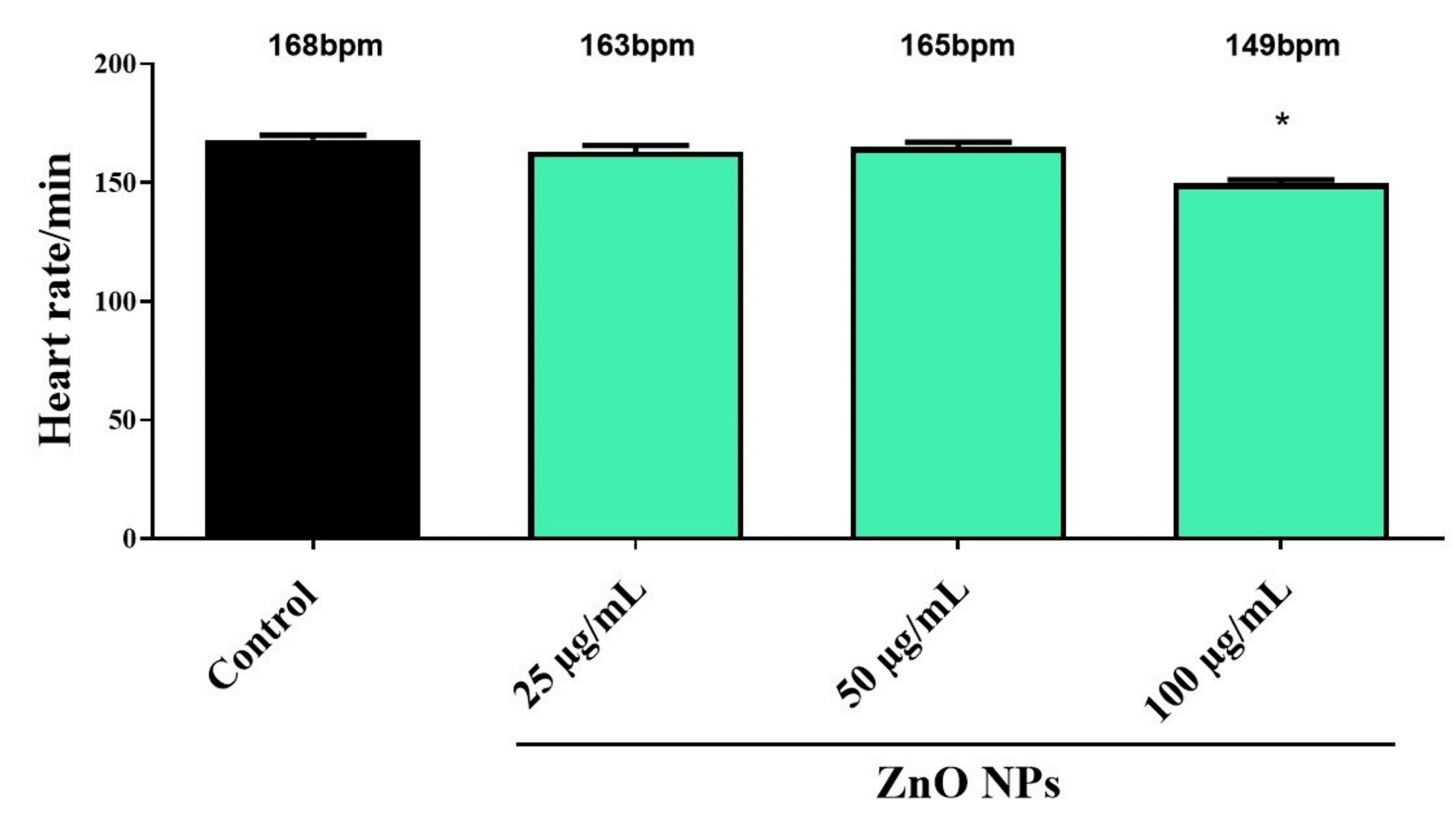
The percentage of heart rate of zebrafish embryo treated with various concentrations of synthesized ZnO NPs. The * (p < 0.05) indicates the significant difference between the control and intervention groups.

The heart rate was calculated by observing the zebrafish under the microscope. The heartbeat rate was calculated as heartbeat/min (bpm) and is shown in Figure 2 for Zebrafish embryos exposed to various concentrations of ZnO NPs. A control group had a heart rate of 168 bpm, whereas groups treated with ZnO NPs had a decreased heart rate. A proportional reduction in heart rate was observed at all concentrations when ZnO NPs were added, with 161 bpm at 25 µg/mL, 163 bpm at 50 µg/mL, and 151 bpm at 100 µg/mL. In addition, the results indicate that ZnO NPs have a dose-dependent effect on heart rate, with greater reductions occurring at higher concentrations.

Effects on hatching rate

**Figure 3:**
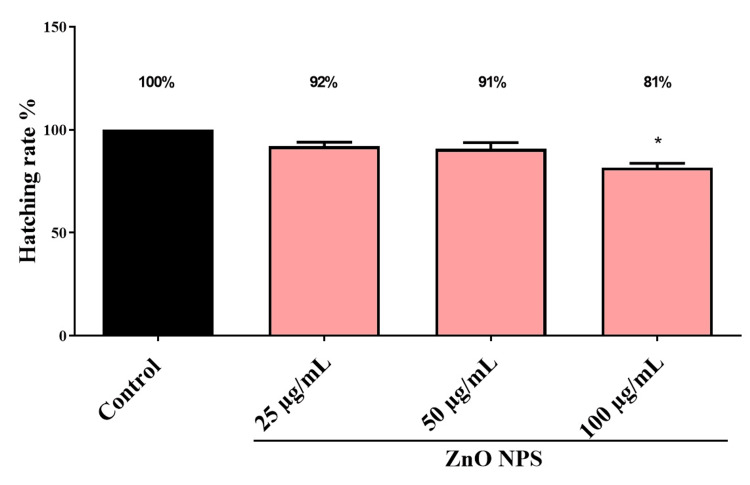
The percentage of hatching rate of zebrafish embryo treated with various concentration synthesized ZnO NPs. The * (p < 0.05) indicates the significant difference between the control and intervention groups.

**Figure 4:**
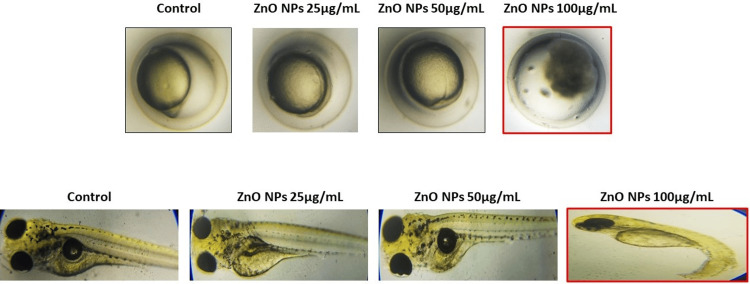
The photomicrographs for the hatch rate and mortality rate of zebrafish embryos at various concentrations of synthesized ZnO NPs. The morphological toxicity condition was observed in the ZnO NPs 100 µg/mL.

It has been shown that ZnO NPs delayed hatching in zebrafish based on their dose. No toxic effects represent a 100% successful hatching rate. As shown in Figure 3, the control group had a hatching capacity of 100%, while ZnO NPs significantly reduced the hatching capacity of zebrafish embryos to 100 µg/mL. Using a novel polyherbal ICM loaded with ZnO NPs, the study demonstrated that different medication concentrations can influence the heart rate, hatching, and mortality rate of zebrafish embryos in a dose-dependent manner. The results of the study are outlined in Figure 4.

## Discussion

In endodontics, nanoparticles can be used as irrigants, ICM, or root canal sealers because of their smaller size and more compact structure, which gives them a larger surface area that can absorb other medications and allows for faster and greater penetration into the dentinal tubules. Kishen et al. [17] observed that ZnO NPs reduced *Enterococcus faecalis* biofilm, but its effectiveness depended on concentration and interaction time. ZnO NPs are believed to release Zn+ ions that can cause high cytotoxicity as well as environmental hazards [18 - 20]. The presence of phytochemicals on green synthesized nanoparticles gives them biological compatibility and a benign nature. Plants, such as *Azadirachta indica* and *Solanum xanthocarpum*, possess a wide range of biologically active compounds that make them potential antibacterial plants. As Spitsbergen et al. stated [21], the zebrafish is an excellent animal model for studying toxicology. Hence, the present study utilized green synthesized ZnO NPs from *Azadirachta indica* bark and *Solanum xanthocarpum* seed extract and tested their toxicity on zebrafish embryos. Based on Hsu et al.'s [22] observation, the cardiovascular, nervous, and digestive systems of zebrafish are similar to those of mammals in many aspects. Zebrafish have lately been promoted by the National Institutes of Health (NIH), USA, as a model organism for disease investigation [23]

In the current investigation, green synthesized ZnO NPs exposed to zebrafish embryos show dose-dependent cytotoxicity in terms of survival rate, hatchability, and heart rate. According to Lee et al. [8], zinc concentration reduction in bulk solutions correlated well with toxicity reduction. Since ZnO and TiO2 have a band gap that is quite close, Brun et al. [24] speculated that the toxicity of ZnO NPs may be comparable to that of TiO2 NPs (3.3 eV).

Zebrafish embryos were exposed to different concentrations of green synthesized ZnO NPs (25, 50, and 100 µg/mL) to evaluate toxicity. The results revealed alterations in the hatching rate compared to controls. Hatching delays were observed at the highest concentrations (100 g/mL) of ZnO NPs, which showed an 80% reduction in hatching rate, but at lower concentrations of 25 g/mL, <5% reduction was seen. The results of our study confirm previous reports, which suggest that ZnO NPs have a dose-dependent inhibitory effect on embryo hatching [25]. Based on a comparison between ZnO NPs and the control suspension, Xiaoshan et al. [25] found that the suspension containing about ≤0.5 mg·L^-1^ of particles did not affect zebrafish embryo hatching, but the toxicities increased with particle concentration >5 mg·L-1. A previous study stated that the hatching retardation of zebrafish embryos might be due to disturbance of the hatching enzyme (proteases) and hypoxia induced by ZnO NPs [26].

There is a direct correlation between the level of toxicity caused by different concentrations of nanoparticles and the survival rate of the zebrafish embryo. At higher concentrations, toxicity levels reached their maximum. ZnO NPs at a concentration of 100 µg/mL, resulting in a notable decrease in the survival rate of the embryos. On the contrary, when the concentration was reduced to 25 µg/mL, the survival rate remained unchanged, similar to the control embryos. As reported by Ramachandran et al. [26], there were no differences in zebrafish mortality between the control and lower concentrations of AgNPs (15.5 and 18.6 μg/L) and AuNPs (9.7 and 19.4 mg/L) in the treated groups. Zhou et al. [27] found that zebrafish lying on the bottom of the aquarium and having an increased respiratory rate were signs of toxicity at higher concentrations of ZnO NPs. As observed by Xiong et al. [28], zebrafish mortality was increased more markedly by ZnO NP exposure than by bulk ZnO exposure. Prior studies have shown that increased mortality from ZnO NPs was related to mouth-gaping behavior, resulting in increased ZnO NP uptake in zebrafish larvae. As shown in Figure 4, there were developmental defects, including tail deformity, spinal curvature, and ulcerations induced by the highest concentration of 100 g/L. Similar results were reported by Zhao et al. [29], who found that exposure to ZnO NPs (100 mg/L) caused morphological defects such as spine curvature and tail deformity. The results of the embryo toxicity test carried out by Bai et al. [23] showed that ZnO NPs delayed the hatching of embryos (1-25 mg/L), decreased the size of the larval body, and resulted in deformity of the tail after 96 hours of exposure.

In light of the study's findings, the heart rate of the fish model seemed to decrease at higher concentrations. Caparucci et al. [30] found a dose-dependent decrease in heart rate at 100 and 200 mg/L compared to the control group. If the heart rate is reduced, the blood flow is slowed, and this may result in the muscles being depleted of glucose, which may lead to a lower muscular response and a lower hatching rate since there is less glucose in the muscles. The green synthesized ZnO NP suspensions, however, showed no significant toxicities on zebrafish embryos at low concentrations. To determine the developmental toxicity of green synthesized ZnO NP, further experiments with long-term exposure studies in vitro and in vivo models are necessary to assess the potential chronic effects and also to identify other potential influencing factors on their toxicity.

Within the limitations of this study, it can be concluded that further clinical trials are still required to fully comprehend the manifestation of these unique intracanal polyherbal ZnO NPs in a clinical setting in order to evaluate their effectiveness in routine endodontic therapy.

## Conclusions

Our research has enabled the development of a novel ICM based on ZnO NPs loaded with polyherbal extracts (*Azadirachta indica* and *Solanum xanthocarpum*), which were tested on zebrafish embryos for their cytotoxic effects. Concentration-dependent toxicity of ZnO NPs was demonstrated affecting heart rate, hatch rate, and mortality rate significantly. The results of this study suggest that this novel ICM with optimized concentrations may be relatively safe for endodontic use.
